# How we teach children with asthma to use their inhaler: a scoping review protocol

**DOI:** 10.1186/s13643-020-01430-6

**Published:** 2020-08-11

**Authors:** Patrick McCrossan, Orla Mallon, Michael D. Shields, Dara O’Donoghue

**Affiliations:** 1 Department of Respiratory Paediatrics, Royal Belfast Hospital for Sick Children, Belfast, UK; 2 Queen’s University Belfast School of Medicine, Dentistry and Biomedical Sciences, Belfast, UK

## Abstract

**Background:**

One reason that asthma remains poorly controlled in children is poor inhaler technique. Current guidelines recommend checking inhaler technique at each clinical visit. However, they do not specify how best to train children to mastery of correct inhaler technique. Currently, many children are simply shown how to use inhalers (brief intervention) which results in less than 50% with correct inhaler technique. The aim of this scoping review is to explore published literature on teaching methods used to train children to master correct inhaler technique.

**Methods:**

This scoping review will follow the Arksey and O’Malley framework and the Joanna Briggs Institute guidelines. We will search (from inception onwards) MEDLINE, Embase, Scopus, Web of Science, CINAHL and the Cochrane library. We will include quantitative studies (e.g. randomised controlled trials, cohort studies and case-control studies), published from the year 1956 to present, on teaching the skill of inhaler technique to children with asthma. Two reviewers will complete all screening and data abstraction independently. Data will be extracted onto a data charting table to create a descriptive summary of the results. Data will then be synthesised with descriptive statistics and visual mapping.

**Discussion:**

This scoping review will provide a broad overview of currently used educational methods to improve inhaler technique in children with asthma. The analysis will allow us to refine future research in this area by focusing on the most effective methods and optimising them.

**Systematic review registration:**

Open Science Framework (osf.io/n7kcw).

## Background

Asthma remains poorly controlled for many children [1]. Regular inhaled corticosteroid (ICS) should provide control for the vast majority of these children [2, 3].

Effective delivery of drugs to the lungs relies on patients using their inhalers correctly [4]. However, inhaler technique, particularly in children, is generally poor [5, 6]. This results in failure to deliver an adequate dose of ICS to the airways [5]. Failure to deliver ICS to attenuate chronic airways inflammation is associated with poor asthma control, increased asthma attacks and increased risk of death [6, 7]. In a retrospective chart review of 142 children with uncontrolled asthma, 7.7% found to have poor inhaler technique as the underlying cause [6].

Inhaler technique has not improved over the last four decades despite major pharmaceutical company investment into easy to use inhaler devices [8]. There are many available guidelines on how to effectively use an inhaler; for example, Asthma UK has instructional videos for all of the commonly prescribed inhalers [9]. However, to the best of our knowledge, there are not yet any guidelines on how to train a child to master the technique of using their inhaler.

Current guidelines recommend checking inhaler technique at each clinical visit [10] at which point many children are simply shown how to use their inhaler (brief instruction) which results in less than 50% with correct inhaler technique [11]. However, it is not known how best to teach children to master the correct technique.

The aim of this study will be to review published literature on teaching the skill of inhaler technique to children with asthma. The specific objectives are as follows:

1. To identify what different educational methods and approaches have been used to teach the skill of inhaler technique in children.
2. To consider whether these methods need to be tailored according to the age of the child.
3. To describe methods suitable for use in a children’s asthma clinic.
4. To determine how the effectiveness of these methods is evaluated.

## Methods

The present protocol has been registered within the Open Science Framework platform (osf.io/n7kcw).

The present study protocol is being reported in accordance with the reporting guidance provided in the Preferred Reporting Items for Systematic Reviews and Meta-Analyses Protocols (PRISMA-P) statement [12] and the PRISMA extension for Scoping Reviews (PRISMA-ScR). The scoping review conduct will be guided by methods and framework of Arksey and O’Malley [13], subsequently adapted by Levac et al. [14] and Colquhoun et al. [15] and the Joanna Briggs Institute guidelines [16]. The reporting of methods and results of the scoping review will be presented using the PRISMA-ScR [17].

The above process of performing a scoping review will involve the following stages:

1. Define the research question.
2. Identify relevant published literature.
3. Select which studies to analyse.
4. Extract and chart the data collected from these studies.
5. Report and disseminate the results.

### Research question

‘What is known about educational interventions for children with asthma to facilitate the mastery of inhaler technique?’ However, due to the iterative nature of a scoping review, the research question or the search terms may alter following a piloting process.

### Identifying relevant published literature

#### Information sources and searches

An initial pilot exercise will be undertaken using the online database MEDLINE before proceeding to a complete search of all available databases. We plan to review 10 publications of varying styles from this initial piloting exercise. The purpose of the pilot is to ensure that we have chosen the most suitable search terms by considering the different concepts that each of these articles could be mapped to.

Once the pilot is complete and consideration of new search terms is made, a full search of MEDLINE will be completed followed by a full search of the subsequent chosen online electronic databases (Embase, Scopus, Web of Science, CINAHL and the Cochrane library) from inception onwards. These databases will provide a comprehensive list of the appropriate literature across a range of interdisciplinary fields. A search of other grey literature (including Google Scholar) and discussion with a group of paediatric asthma specialists will also be undertaken to handpick any further highly relevant studies. At this point, we will record the total number of citations yielded by these search terms from each of the databases.

Under the guidance of the Queen’s University Belfast medical librarian, a search strategy will be developed. We will include a range of terms and keywords related to the research question, such as “asthma”, “wheeze”, “children”, “paediatrics”, “inhaler technique”, “educational intervention”, “training” or “education”. A draft search for MEDLINE is provided in Additional file 2.

### Selecting studies

#### Eligibility criteria

We will include studies published since the year 1956 (when the pressurised metered-dose inhaler (pMDI) was first introduced to clinical practice [18]). We will include randomised control trials, case-control studies and cohort studies which investigate methods used to teach inhaler technique to children (patients under the age of 18). In order to consider a full breadth of knowledge in this subject area, we will include conference abstracts, presentations and scholarly information that has not necessarily been peer reviewed. We will include studies in which the teaching of inhaler technique was provided by doctors, nurses, pharmacists and physiotherapists. We do not wish to be ‘device specific’ and so will include studies of metered-dose inhalers and dry powder inhalers, with and without the use of spacer devices. We will include studies which have taken place in the emergency department, the out-patient department, the hospital ward and/or the community as these are all areas where inhaler technique teaching occurs.

We will exclude studies of the use of nebulised therapies as this involves a different technique and is a more passive procedure. Studies which involve adult participants only will be excluded. We will include studies which include adults and children only if the children’s data has been presented separately and can therefore be reviewed as a study of children with asthma. Publications not in English will be excluded.

#### Screening

Selection of studies will then be performed in two stages. There will be an initial screening of all publications based on the title with further screening based on the abstract, to ensure they fulfill the eligibility criteria (P McCrossan). Documents not meeting the eligibility criteria will be excluded from the full-text analysis.

The remaining publications will then be read by two reviewers, from the research team (P McCrossan, O Mallon and D O’Donoghue). These two reviewers will independently analyse the content included in the full-text articles. If there is any case of uncertainty, the text will be re-evaluated by a third independent reviewer (MD Shields). The final search results will be exported into Endnote™ at which point all duplicates will be detected and eliminated.

A flow chart showing details of studies included and excluded at each stage of the study selection process will be provided.

### Extracting and charting the data

A data extraction form will be designed (see Additional file 3) and used to extract equivalent information from each study report. This data extraction form may be refined following a review of this process with the first 2 or 3 studies, purposively chosen as diverse (in keeping with the iterative nature of the scoping review methodology) [16]. Subsequently, each of the included studies will be abstracted by two reviewers, independently, and potential conflicts will be resolved through discussion. Authors of primary publications will be contacted for data clarifications or missing outcome data, as necessary. Information of interest will include the following:

- Study characteristics: study design, year of publication, journal, year (or period) of study conduct, sample size, country, setting, aim and other fields to capture data relevant to the assessment of study methods.
- Participant characteristics: age (e.g. mean with standard deviation, range), gender (e.g. percentage of female participants), definitions of asthma (according to standard diagnostic criteria).
- Inhaler characteristics: name, design (pressurised metered-dose inhalers and dry powder inhalers) and use of inhaler adjunct devices such as spacers.
- Technique training characteristics: definitions of the educational intervention content and delivery (as described by study authors), including who was providing the inhaler training.
- Outcomes: outcome measures related to how the effectiveness of the technique training is evaluated.
- Concept: the research group will come to a consensus of what the overall ‘concept’ of each study was.

### Data synthesis

The data will be summarised in a descriptive format (narrative synthesis), in visual format (mapping summary) and tabular form (numerical summary). The strategy for data synthesis entails the use of qualitative methods to categorise the educational interventions based on the treatment modality as well as subgroup diagnosis and age group, e.g. preschoolers aged ≤ 5 years old, as they are often distinct in the literature as regards phenotype but also present different challenges as regards inhaler technique compliance [19]. Any commonalities between studies will be synthesised and presented. A qualitative descriptive synthesis of data will be undertaken in mapping the intervention modalities.

## Discussion

Poor or incorrect inhaler technique remains a major cause of persistent poor asthma control in children. It is relatively common for children prescribed inhalers only to be shown (with a brief intervention) how to use their inhaler rather than trained to mastery. Effective teaching methods that can be used at a children’s asthma clinic that result in more children mastering their inhaler remain an under-researched area. A scoping review of the literature is an important first step in addressing the clinical need to improve inhaler technique for children with asthma. Optimising this fundamental aspect of asthma management will improve patient outcomes.

At intervals during the data extraction and charting process, charted data will be compared and discussed to ensure consistency between reviewers and to enable iterative reflection on emerging themes and categories. During the review process, any amendments to the protocol deemed necessary by the team will be recorded in the master protocol document with the reason for amendment noted on file.

We acknowledge that this study will be limited to those publications in the English language; however, the broad nature of the research question will still allow us to capture a significant proportion of the available literature. It is not the intended purpose of a scoping review to formally evaluate the quality of the evidence, and so, this study is limited to descriptive accounts of the included publications and variations in their quality. There is the potential for selection bias which will be mitigated, to some extent, by multiple members of the research team independently reviewing the data.

The results of this scoping review will be disseminated through peer-reviewed publications and presented at international conferences targeting an audience interested or involved in paediatric asthma.

This scoping review will provide a broad overview of currently used educational methods to teach the skill of inhaler technique to children with asthma, the analysis of which will allow us to refine future research in this area by focusing on the most effective methods and optimising them.

## Supplementary information

**Additional file 1.** Preferred Reporting Items for Systematic reviews and Meta-Analyses extension for Scoping Reviews (PRISMA-ScR) Checklist.

**Additional file 2.** Draft search strategy to be used for MEDLINE.

**Additional file 3.** Example of data extraction chart.

## Data Availability

Not applicable.

## Abbreviations

ICS: Inhaled corticosteroids
pMDI: Pressurised metered-dose inhaler
DPI: Dry powder inhaler
