# Supplementary material for: How we teach children with asthma to use their inhaler: a scoping review protocol
Source: Syst Rev. 2020 Aug 11;9:178. doi: 10.1186/s13643-020-01430-6 (PMC7422595; doi:10.1186/s13643-020-01430-6)
Supplement: Supplementary file 2 — Additional file 2. Draft search strategy to be used for MEDLINE. [file 13643_2020_1430_MOESM2_ESM.docx]

**Draft search strategy to be used for MEDLINE**

1. Childhood asthma
2. Childhood wheeze
3. Asthma
4. Wheeze
5. **1 or 2 or 3 or 4**
6. Children or Child
7. Paediatrics or Pediatrics
8. **6 or 7**
9. Inhaler technique
10. Metered dose inhaler
11. Dry powder inhaler
12. Inhaler management
13. Inhaler method
14. Administration, Inhalation
15. **9 or 10 or 11 or 12 or 13 or 14**
16. Patient education as Topic/ or educational intervention
17. Video games/ or Compuer-Assisted Instruction/ or serious games or Games, experimental
18. Patient instruction
19. Instruction
20. Health education
21. **16 or 17 or 18 or 19 or 20**
22. **5 and 8 and 15 and 21**

**Limits:**

Published in English language

Publication year; 1956-present (2020)
