# Supplementary material for: How we teach children with asthma to use their inhaler: a scoping review protocol
Source: Syst Rev. 2020 Aug 11;9:178. doi: 10.1186/s13643-020-01430-6 (PMC7422595; doi:10.1186/s13643-020-01430-6)
Supplement: Supplementary file 3 — Additional file 3. Example of data extraction chart. [file 13643_2020_1430_MOESM3_ESM.docx]

**Additional file 3**

Example of data extraction chart

| Author | Year | Country of origin | Aims | Studied population | Methodology | Intervention type | Who provided the inhaler training? | Concept | Measured outcomes | Key findings |
| --- | --- | --- | --- | --- | --- | --- | --- | --- | --- | --- |
|  |  |  |  |  |  |  |  |  |  |  |
|  |  |  |  |  |  |  |  |  |  |  |
